# An overview of advancements in closed-loop artificial pancreas system

**DOI:** 10.1016/j.heliyon.2022.e11648

**Published:** 2022-11-14

**Authors:** Doni Dermawan, Muhammad Abiyyu Kenichi Purbayanto

**Affiliations:** a Applied Biotechnology, Faculty of Chemistry, Warsaw University of Technology, Warsaw, Poland; b Faculty of Materials Science and Engineering, Warsaw University of Technology, Warsaw, Poland

**Keywords:** Closed-loop artificial pancreas, Glucose sensor, Insulin, Type 1 diabetes

## Abstract

Type 1 diabetes (T1D) is one of the world's health problems with a prevalence of 1.1 million for children and young adults under the age of 20. T1D is a health problem characterized by autoimmunity and the destruction of pancreatic cells that produce insulin. The available treatment is to maintain blood glucose within the desired normal range. To meet bolus and basal requirements, T1D patients may receive multiple daily injections (MDI) of fast-acting and long-acting insulin once or twice daily. In addition, insulin pumps can deliver multiple doses a day without causing injection discomfort in individuals with T1D. T1D patients have also monitored their blood glucose levels along with insulin replacement treatment using a continuous glucose monitor (CGM). However, this CGM has some drawbacks, like the sensor needs to be replaced after being inserted under the skin for seven days and needs to be calibrated (for some CGMs). The treatments and monitoring devices mentioned creating a lot of workloads to maintain blood glucose levels in individuals with T1D. Therefore, to overcome these problems, closed-loop artificial pancreas (APD) devices are widely used to manage blood glucose in T1D patients. Closed-loop APD consists of a glucose sensor, an insulin infusion device, and a control algorithm. This study reviews the progress of closed-loop artificial pancreas systems from the perspective of device properties, uses, testing procedures, regulations, and current market conditions.

## 1 Introduction

Diabetes has become a worldwide health issue and is included as one of the top 10 causes of death in adults. About 463 million (9.3%) adults are recently living with diabetes worldwide. This number is estimated will be rosed until 700 million (10.9%) of the world population. The burden caused by diabetes increased not only from the healthcare aspect but also from the global health expenditure that reached about United States dollar (USD) 760 billion by 2019. The healthcare expenditure is forecasted to be USD 825 billion by 2030, and it will be increased to USD 845 billion by 2045 [1].

Type 1 diabetes (T1D) is one of three main types of diabetes alongside type 2 diabetes (T2D), and gestational diabetes mellitus (GDM) [2]. An amount of 1.1 million children and those aged under 20 years old have T1D by 2019 [1]. T1D is an autoimmune-associated health problem and destruction of pancreatic β cells for producing insulin [3]. Insulin is essential for the human body to reduce the concentration of blood glucose then the energy could be generated [4]. In the T1D patient, there is a significant loss of insulin and amylin secretion. Despite the tiny percentage of monogenic T1D patients, recently, there has been no therapy to overcome the root cause of diabetes. The available treatments are the only option to maintain blood glucose within the desired normal range (70–180 mg/dL) [5]. The discovery of exogenous insulin around 100 years ago has a notable positive progression to prolong the short lifespan of T1D patients. Various types of treatment have been carried out such as insulin injection and continuous insulin infusion using the insulin pump [6].

Multiple daily injection (MDI) treatment with long-acting insulin can be administered once or twice a day to T1D patients to meet the requirement of basal needs. On the other hand, MDI with rapid-acting insulin is given to meet the meal insulin needs and refine the hyperglycemia condition [7]. Another way to provide exogenous insulin for T1D patients is the insulin pump, a small external device that can continuously deliver amounts of rapid-acting insulin. Insulin pumps consist of a tube that connects the reservoir to the infusion site. Insulin pumps are more favorable than MDI for basal and bolus delivery purposes. Insulin pumps can deliver a very small amount of insulin to 0.01 units compared to syringe injections that only 0.5 units for the smallest amount. Besides, insulin pumps can provide multiple doses a day without the discomfort caused by injections to individuals with T1D [8]. From the development perspective, patch pumps for Insulin are newly developed to optimize the accuracy and flexibility of insulin delivery at lower costs. The insulin patch pump is a fully flexible device for performing the most complex regimens for insulin-treated diabetic patients. This device works on the principle of electromechanical and has a mechanical pump with an electronic controller. The mechanism of action of this patch pump is based on a needle inserter that inserts a metal needle measuring about 4 mm 30 ga into the patient's subcutaneous tissue. The device has a syringe-shaped plunger spring containing silicone oil [9].

T1D patients have also monitored their blood glucose level alongside insulin, replacing treatment by employing the continuous glucose monitor (CGM). CGM is an electrochemical sensor placed subcutaneously to measure the interstitial fluid of glucose levels. The sensor is linked to the transmitter, which transfers the current of blood glucose to a portable device every certain time and it alerts the users when the blood glucose level exceeds the hypoglycemia or hyperglycemia thresholds [10]. However, this CGM has several drawbacks, such as the sensor needs to be replaced after being inserted under the skin for seven days, and it needs to be calibrated (for some CGMs) multiply each day to maintain the accuracy and precision levels of measurement [11].

The mentioned treatments and monitoring devices produce a lot of workload for maintaining blood glucose levels in individuals with T1D. This issue has to be solved to improve the goals of therapy, improve patient comfort and compliance, and also improve cost efficiency. It also needs automated insulin delivery and monitoring to prevent short-term and long-term complications caused by T1D and minimize its daily burden.

## 2 Method

A structured literature search was employed using PubMed after identification of free-text terms and MeSH terms, for searches within titles and abstracts relating to the four components of the search: closed-loop artificial pancreas, glucose sensor, insulin, and type 1 diabetes. The search query was: (((closed-loop artificial pancreas) AND (glucose sensor)) AND (insulin)) AND (type 1 diabetes). The search was conducted on January 16, 2022, and updated on June 08, 2022. This search yielded 172 articles, which consisted of 57 clinical trials, 2 meta-analyses, 47 randomized controlled trials, 46 reviews, and 4 systematic reviews. Search results not related to closed-loop artificial pancreas for type 1 diabetes were excluded (12 articles). The remaining articles from the exclusion system were then read, understood, and interpreted to produce reviews relevant to closed-loop artificial pancreas. Furthermore, several studies on FDA-based regulation were also included.

## 3 Description of the device properties

One of the most widely used for managing blood glucose in T1D patients is the artificial pancreas. The artificial pancreas device (APD) is a closed-loop control system of blood glucose in individuals with diabetes. This closed-loop APD consists of a glucose sensor, an insulin infusion device, and a control algorithm. The early approved APD by the Food and Drug Administration (FDA) in the early 1970s is Biostator™ that by Miles Labs for managing insulin-dependent diabetes patients [12]. APD can be classified by its configuration, such as a single-hormone APD, which only delivers insulin and a dual-hormone APD, which delivers insulin and glucagon [13]. On the other hand, Based on the automation level, closed-loop APD can be categorized into three different generations. First-generation APD consists of three main stages, including (1) insulin pump turns off when there is the response from user to low glucose alarm; (2) alarm for hypoglycemia that followed by reduction of insulin delivery, and (3) similar to 2nd stage with a higher threshold. The second-generation consists of stage (4) hybrid closed-loop at all times with bolus-assisted for mealtime and stage (5) fully-automated insulin closed loop. The third generation comprises stage (6) fully-automated multi hormones (insulin added with glucagon or amylin) closed-loop APD [14].

An automated closed-loop APD is defined as an externally worn medical device system that consists of three main functions, including (1) insulin delivery carried out by an insulin infusion pump; (2) monitoring glucose levels carried out by a CGM, and (3) control center function carried out by a digital controller [15]. Several types of insulin infusion pumps are available on the market including tubeless patch pumps, automated delivery of insulin with a hybrid closed-loop system, and multiple closed-loop systems. There is also an AndroidAPS-assisted system using Bluetooth commands in a smartphone [16]. CGM improvement is proportional to sensor reliability, accuracy, and feature development. The main sensors are based on electrochemical glucose oxidase reactions to determine blood glucose levels [17]. A control algorithm system is employed for maintaining target blood glucose variables. The control strategies include (1) proportional, integral, derivative controller; (2) model predictive control; and (3) fuzzy logic [16].

**Figure 1:**
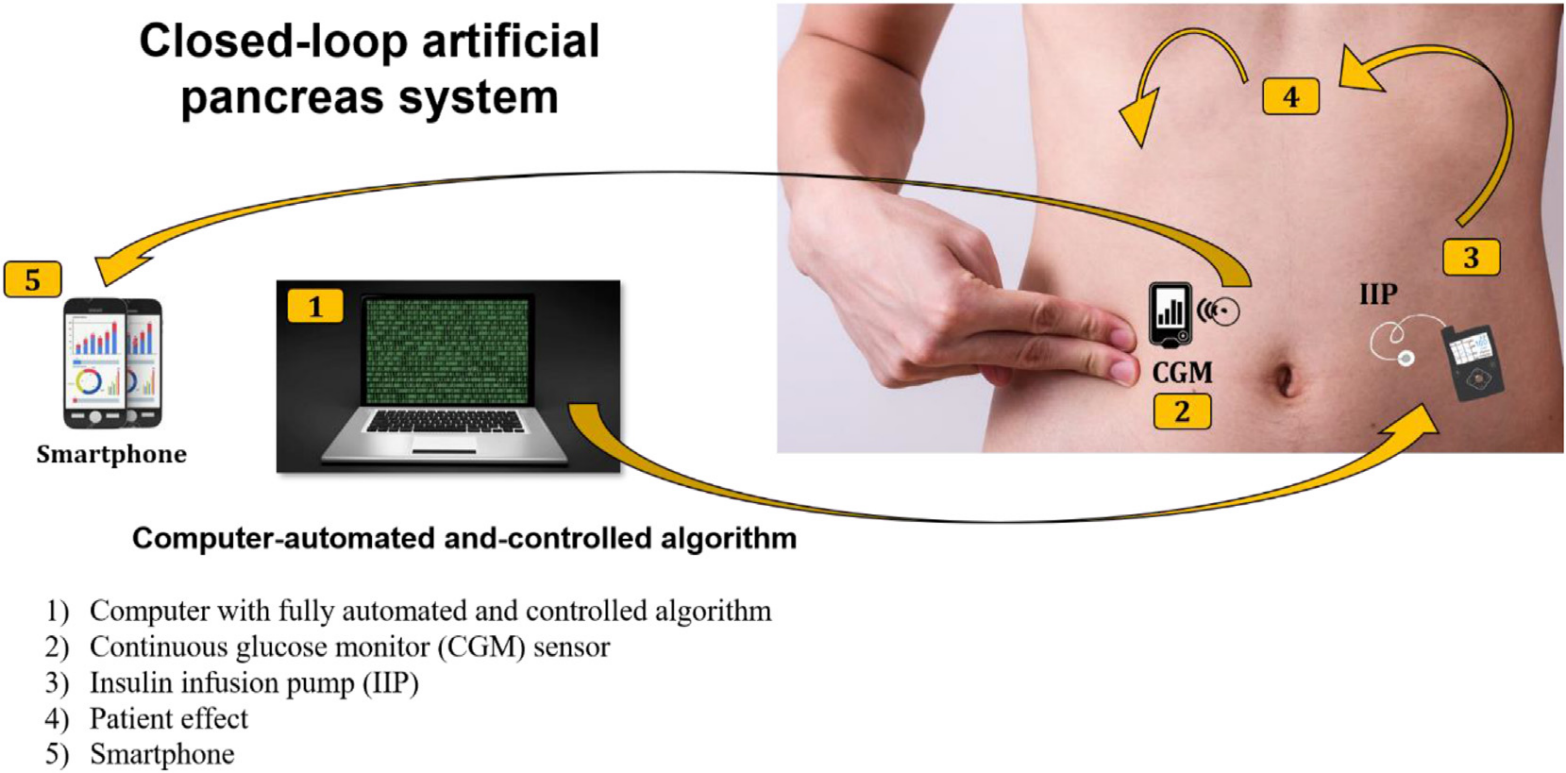
Schematic implementation of closed-loop artificial pancreas systems. (1) A software installed in an external controller that obtains information from the CGM and executes a series of mathematical calculations. Then, the controller will send dosing instructions to the insulin infusion pump. (2) CGM worn subcutaneously delivers the data about interstitial glucose concentrations to a smartphone. (3) As the final step, the amounted insulin delivery is regulated in real-time by the control algorithm.

In general, the recent system consists of four main components that integrated each other as depicted in (Figure 1):

### 3.1 APD automated and controlled algorithm

The control algorithm is one of the vital components of APD. It regulates the correct insulin injection rate based on CGM's measured blood glucose level. The usage of a control algorithm could hinder the event of hypoglycemia and decrease time in hyperglycemia. The desired control algorithm should have the ability to overcome the problem in glucose-insulin dynamics, such as the delay associated with subcutaneous insulin infusion, time-varying dynamics, inter-individual variability, and strong non-linear phenomena [18]. Additionally, control algorithms have an essential role in closed-loop therapy by facilitating the personalization and adaptation of the AP system [19]. Several algorithms have been tested *in-silico* and clinically over time and improved over the years [20].

#### 3.1.1 Model predictive control

The model predictive control (MPC) model utilizes the prediction model to predict the effects of proposed manipulated input changes on the desired output [21]. Note that MPC is not a single exact algorithm, it is instead composed of several algorithms tailored based on the desired goals. MPC is considered one of the most efficient control strategies developed in recent years. MPC could solve the problem in the presence of significant disturbances (i.e., meals and physical exercises) and delays in the effect of meals and insulin absorption [22]. In addition, MPC offers high flexibility in controlling parameters for each patient [23]. However, to utilize the MPC algorithm, one must carefully implement the mathematical models of glucose-insulin interaction. Several mathematical models have been proposed by researchers to be implemented in MPC. Generally, the models can be classified as linear and non-linear models. The non-linear model is viewed as a suitable mathematical model for glucose-insulin interaction due to this system shows a high non-linearity nature. However, the non-linear model is not applicable for portable APD as it demands high computational performance. Currently, the development of the MPC control algorithm tries to adapt customized linear models that can tailor to individual patient needs leading to better glucose control [24]. Messori et al. presented three different customized linear models which could improve glucose control performance, including the carbohydrate ratio (CR)-based model, non-parametric model, and constrained optimization model [24]. However, the proposed models were only tested *in silico*.

MPC has been successfully used in outpatient clinical studies and has shown promising results [25, 26, 27]. For instance, a multicenter study conducted by Brown et al. demonstrated that closed-loop control (CLC) utilizing the MPC model could improve glucose control with fewer hypoglycemia events overnight [27]. What is more, those beneficial CLC outcomes were also observed during the daytime. Furthermore, Messori et al. conducted the study using run-to-run (R2R) adaptive wearable AP under free-living conditions [26]. Here, they utilized the MPC linear model coupled with real-time corrections to the basal insulin and the meal bolus. The results show that the R2R-AP significantly improved glucose control of T1D patients during the night. Interestingly, the equivalent control performance was maintained during the day in a one-month trial under free-living conditions.

#### 3.1.2 Proportional-integral-derivative

Proportional-integral-derivative (PID) control is based on the principle of controlling a single target quantity *via* a control-feedback calculated as a simple summation of the proportional, integral, and derivative term. In diabetes therapy, the PID algorithm adjusts insulin delivery by assessing glucose excursion from three perspectives, i.e., deviation from target glucose (proportional term), area under the curve between measured and target glucose (integral term), and the rate of change in measured glucose (derivative term) [28]. PID is known as the simplest way to design a robust control system for the patient with TD1. Pinsker et al. compared the performance between MPC and PID algorithm and concluded that MPC performs better than PID as it achieved nearly 75% in the target range, including the unannounced meal [29]. However, this finding was highlighted by Steil, in which he justified that the results of Pinsker et al. cannot be extended to include all available PID algorithms [30].

#### 3.1.3 Fuzzy logic

Unlike MPC and PID, which rely on mathematical models in describing human glucoregulatory systems for interpretation of glucose data and insulin administration, fuzzy logic (FL) uses glucose management parameters to determine the insulin doses. The introduction of FL algorithm could give an alternative solution to the problem incorporating various physiological parameters such as illness and stress. As stated previously, FL relies on predetermined glucose parameters by an expert diabetes clinician. Three terms are tailored (glucose level, glucose slope or rate, and glucose acceleration) to cover all possible dosing situations that a person with diabetes would normally encounter [31]. From the study by Mauseth et al., using an FL controller could be an alternative to an MPC-based controller as a component of a closed-loop insulin delivery system [31].

Recent advanced closed-loop artificial pancreas system based on FL controller employs the novel “*MD-Logic Artificial Pancreas algorithm*” as a fully digitalized and advanced hybrid closed-loop system to control user glucose levels. It can change basal insulin and provide auto-correcting bolus in real-time conditions [32]. This process relies on the dynamics of the glucose sensor readings and the information from the insulin infusion pump. Furthermore, the effectiveness of the MD-Logic algorithm has been validated in many clinical studies. MD-Logic algorithm of advanced closed-loop artificial pancreas system is integrated into the Android and iOS platforms.

### 3.2 Continuous glucose monitor (CGM)

A wearable device that lets the users take a look at their glucose readings every single minute in real-time. CGM contains three main elements such as (1) sensor: a tiny wire inserted under the stomach's skin that measures blood glucose every minute; (2) receiver: display the obtained data from the sensor, and (3) transmitter: a wireless component of sensor that delivers blood glucose level to the receiver. Each CGMs have their calibration standard.

#### 3.2.1 From self-blood glucose measurement to continuous glucose measurement

The discovery of insulin in the early 1920s brought a significant breakthrough in the therapy and the prognosis of diabetic patients. Insulin therapy has an integral part of diabetes treatment. Insulin plays a role in managing blood sugar and preventing diabetes complications by keeping blood sugar in the target range (normal or near-normal glycemia). It is known that the normal range of blood sugar ranges from 3.8-5.6 mmol/L. In comparison, hyperglycemia and hypoglycemia events occur when the blood sugar level up above 8 mmol/L and falls below values of 3 mmol/L, respectively [33]. Therefore, in order to keep the glucose level in the target range, researchers developed glucose sensing devices for monitoring and measuring glucose levels in physiological fluids both *in-vivo* and *in-vitro*. Besides achieving the glycemic target, glucose monitoring is also crucial for patients in (1) getting the proper treatment on symptoms, (2) assessing response to therapy, and (3) preventing or delaying the progression of complications in patients [34].

For monitoring and assessing their blood sugar at home, patients can use a self-monitoring of blood glucose (SBMG) device. By using SBMG, patients can adjust insulin doses for self-medication and detect hypoglycemia [35]. SBMG device measures patients' blood glucose levels with the assistance of conventional blood glucose meters to measure finger-prick blood samples. SBMG approach is known as a non-continuous method due to the measurement carried out only at a specific point during the day [36]. American diabetes association (ADA) recommends patients with diabetes type 1 to measure their blood glucose three times or more each day [37]. ADA also recommends diabetic patients to use SBMG as it could be a guide to successful therapy and to achieve postprandial glucose targets.

The need for obtaining a real-time blood sugar level and trend to predict a hyperglycemic and hypoglycemic event leads to the development of continuous glucose monitoring (CGM). Nowadays, CGM is becoming a major focus of research in diabetes management. Unlike SBMG, the CGM device has the capability to measure a blood sugar level every 5 min with 288 readings daily [34]. Thanks to CGM development, it can help patients and physicians see a blood sugar pattern and decide when a therapy adjustment is required. A typical CGM device consists of a sensor, electronic processing unit, and display unit. In CGM, a glucose-measuring sensor is placed under the skin in the subcutaneous tissue. Theoretically, the sensors can be placed in any part of the body, but the commonplace to put the sensors are the upper-back arm, the abdomen, or the upper-buttock area [38].

#### 3.2.2 Overview of the developments in blood glucose sensors

The basic principle of current glucose meters is based on electrochemical measurement. Here, a small drop of blood is tested in the electrochemical strips to perform the measurement. Two standard methods are used in electrochemical measurements, i.e., amperometric and colorimetric [39]. Both approaches exploit enzyme strip tests for determining the glucose level. The former measured the electrical current produced from the reaction between glucose and enzyme. The latter measured the color change and intensity of the test strip when the blood sample was exposed to enzyme glucose oxidase [40]. Generally, a glucose monitoring system employs the interaction with the following enzymes: glucose oxidase (GOx), glucose dehydrogenase (GDH), and hexokinase [41, 42]. For self-monitoring purposes, the devices are usually constructed by using GOx and GDH. However, hexokinase is generally used in the laboratory setting as a reference glucose result [43]. This section will give an overview of electrochemical and non-electrochemical-based glucose monitoring devices.

In general, glucose biosensors can be classified into electrochemical-based and non-electrochemical-based biosensors. To date, electrochemical-based biosensors have been developed up to the fourth generation. In the first up to the third generation, GO_x_ and GDH enzymes were mainly utilized as a redox center. In the fourth generation of biosensors, the non-enzymatic approach has been introduced. Here, the non-enzymatic approach could solve several problems faced by conventional sensors. The conventional enzyme-based electrochemical method is known to be susceptible to environmental factors, such as temperature, humidity, and pH value [44]. Several inorganic materials have been successfully employed for fourth-generation biosensors, including carbon nanotubes, reduced graphene oxide [45], graphene oxide [46], nickel microparticles [47], MXene [48], and platinum [49]. However, the critical concerns in realizing these materials as the future glucose sensors are related to the biocompatibility issue and ensuring the deposited materials grow uniformly [50].

Electrochemical-based sensors suffer instability and unsatisfactory accuracy. Therefore, researchers try to seek new solutions to overcome these issues. Non-electrochemical-based biosensors, for instance, optical measurement, were assessed as a promising method in glucose sensor development as it offers fast measurement (the data can be obtained in less than a minute) and is reagent-free [51]. Several optical technologies have been reported for glucose measurement, including near-infrared, mid-infrared, Raman, photoacoustic, fluorescence, and optical coherence tomography. Most optical methods employ light intensity change from nanomaterials or molecular recognition compounds where glucose can bind reversibly [52]. Optical sensors can be placed non-invasively or invasively, but such sensors need to be carefully constructed. Until now, only the fluorescence method has been employed in commercial CGMs. The fluorescence method is considered the most successful optical biosensor so far. Some problems related to miniaturization, cost, and improving signal-to-noise ratio need to be addressed in the future.

#### 3.2.3 Commercial CGM in the market and the technology behind

The market of commercial CGM enjoys rapid growth of market share and it is expected to reach USD 10.36 billion by 2028 [53]. Such a high market development is owing to the capability of CGM to provide and keep track of glucose levels over a designated period. Currently, four companies have become major manufacturers of CGM, i.e., Abbott, Dexcom, Senseonic, and Medtronic. These companies offer different and unique technologies in respect to each other, aiming to give better glycemic control. Currently, in the US market, Abbott offers CGM with the name i.e., FreeStyle Libre and FreeStyle Libre 2. Dexcom introduced G5 mobile and G6, while Medtronic launched Guardian Sensor 3. Interestingly, unlike the other CGMs which use the electrochemical-based sensor, Eversense uses optical-sensors CGM with the market name i.e., Eversense XL.

Medtronic and Dexcom utilize the first-generation glucose sensor with GOx as an oxidoreductase. Dexcom uses two electrodes (working and reference), while Medtronic separates counter and reference electrodes into three electrodes. Abbott used a more sophisticated generation of biosensors i.e., second-generation glucose sensing. From the construction of the electrode, Abbots also uses a three-electrode system as we can see in Medtronic CGM. Here, the osmium complex is used as an electron mediator. One of the key advantages of this second-generation sensor is lower redox potential, making Abbott CGM less prone to interference substances compared to other CGM [54]. Senseonic constructs CGM with different sensing approaches. Instead of electrochemical-based measurement, the device uses an optical-based sensor. Here, the fluorescence method is used by utilizing the interaction between fluorophore-linked divalent boronic acid and glucose.

### 3.3 Insulin infusion pump (IIP)

Based on the instructions sent by the controller, the infusion pump adjusts insulin delivery to the subcutaneous tissue. IIP of advanced closed-loop artificial pancreas system uses rapid-acting insulin such as aspart, glulisine, and lispro since this IIP delivers small amounts of insulin every few minutes, thus the usage of longer-acting insulin is unnecessary.

### 3.4 Intervention effect

The intervention effect is defined as the effect of an advanced closed-loop artificial pancreas system on blood glucose levels. The main goal must be achieved that the blood glucose is controlled properly.

### 3.5 Smartphone

The smartphone acts as a data receiver from CGM that can display the entire blood glucose level and control the insulin dosing.

## 4 The main effect of the usage of the device

Controlling the blood glucose levels within 24 h a day as a target, preventing exercise-induced hypoglycemia, and preventing postprandial hyperglycemia are the main goals of using the ideal closed-loop APD [55]. The main effect of closed-loop APD can be classified into short-term and long-term effects:

### 4.1 Short-term effects

The ideal aim of closed-loop APD is to control meal-related hyperglycemia. However, there is also the biggest risk of the usage of this device which is hypoglycemia due to an over delivery of insulin, excessive physical exercise, and consumption of alcohol [56, 57]. Emergency carbohydrates can be consumed if hypoglycemia cannot be avoided by decreasing insulin delivery or increasing glucagon. Thus, it needs a reliable control algorithm to combine multiple hypoglycemia prevention procedures such as initiation of glucagon when the blood glucose reaches the threshold (50–90 mg/dL), suspension and control insulin delivery, and escalation of alarms to remind the user [58, 59]. However, a study suggested that the short-term effect of using the artificial pancreas system (combination of CGM, insulin pump, and controller) was reducing the HbA1c level by 0.6 percentage units without a possible increase in the patient's risk of developing hypoglycemia [60].

### 4.2 Long-term effects

The ultimate long-term benefit of the usage of closed-loop APD is to prevent diabetes-related complications and prolong the lifespan of individuals with T1D. The long-term complications are retinopathy, cardiovascular disease, and neuropathy. The result of the diabetes control and complication trial showed that a risk gradient per 1 SD (gradient risk) is higher than the HbA1c variable. For instance, a value of 1.3 for a complication of diabetes denotes that the risk elevates by 30% when the HbA1c variable increases by 1 SD [61]. A study conducted in Sweden showed that long-time use of CGM should be reimbursed in combination with an insulin pump if the level of HbA1c > 9.8% (poor glycemic control) or if ≥ 2 severe hypoglycemic events have occurred within 1 year to improve glycemic control in clinical practice (reported considerable non-severe hypoglycemic events in long-time users) [62].

## 5 Necessary tests to be conducted to determine the device's suitability of use

The effectiveness of closed-loop APD must be determined to achieve the main short-term and long-term goals. The effectiveness is affected by two major aspects, including the device specifications and the physiological condition of the users. Based on the device specifications, several key parameters need to be assessed, such as:

### 5.1 Device specifications

#### 5.1.1 Accuracy and precision of CGM

The accuracy and precision of CGM are the essential parameters related to the reliability of measurement. The most recent available CGMs in the market can achieve the mean median absolute relative difference (MARD) of sensor <10%, which is compatible with closed-loop blood glucose control. Based on the *in silico* study, a sensor with MARD ≤10% can determine the decision of insulin dose accurately [63]. Proper calibration and rapid drift of sensor sensitivity also affect the accuracy of CGM. If these parameters are inappropriate, persistent deviations could have occurred, then insulin will be over-delivered, increasing the possibility of hypoglycemia [64].

To avoid hypoglycemia and hyperglycemia events, determining the blood glucose concentration of T1D patients is crucial. CGM is a novel approach to measuring glucose levels compared to conventional capillary blood glucose measurements. However, there are three challenges that we need to resolve to measure blood glucose concentration by CGM accurately, i.e., (1) uncertainty of CGM data due to noise disturbance, (2) CGM accuracy, and (3) time-lag in CGM sensing that may become longer than 10 min [65]. To deal with these CGM issues, various algorithms have been developed. For instance, Bazaev et al. developed an algorithm based on the sigma model or logistic function. The algorithm can predict the blood glucose levels 2 h in advance by utilizing the data from an invasive glucose meter as an initial blood glucose level [66]. Furthermore, Pérez-Gandía et al. proposed the usage of an artificial neural network (ANN) algorithm in conjunction with CGM data [67]. Here, patients' blood glucose level was predicted by using ANN by employing only historical CGM data as an input without the need to use an invasive method for processing the data. The results showed that the ANN algorithm gave a promising result without a significant prediction delay. However, this method has a limitation in predicting a sudden change in glucose levels, such as during meal-intake and physical exercise. Therefore, Facchinetti et al. proposed the smart sensor concept by introducing denoising, enhancement, and prediction module to CGM [68]. The denoising module could reduce the irregularity of CGM measurement and subsequently reduce the number of false hypoglycemic and hyperglycemic events. The enhancement module was shown might improve CGM accuracy. Finally, the prediction module allowed CGM to anticipate hypoglycemic and hyperglycemic events up to 15 min before they occur.

In addition, sensor fault detection and data reconciliation are needed to ensure the CGM sensor gives data with high quality and accuracy. Generally, sensor errors can be divided into two, i.e., hard errors (complete hardware failure) and soft errors (bias, drift, and outlier) [69]. In the case of hard errors, they can be solved by replacing new sensor components and recalibration. On the other hand, soft errors are handled by replacing the erroneous data with automatically estimated values. Hybrid online multi-sensor error detection and functional redundancy (HOMSED&FR) have been tested to overcome sensor errors and the results showed that the proposed method has successfully detected most of the errors and reconciled erroneous reading with the data close to the real value [70]. Here, the HOMSED&FR algorithm was constructed by combining the outlier Kalman filter and a locally-weighted partial least squares regression model. Furthermore, the more complex algorithm (smart multiple-model) for detecting CGM error was developed based on four models: Kalman filter, locally-weighted partial least squares regression model, predictor-based subspace identification, and approximate linear dependency-based kernel recursive least square [71]. In addition, an artificial neural network is used as a voting algorithm to integrate these four different models into one system. The results showed that the smart multiple-method could detect most CGM sensor faults such as missing signal, drift change, signal stuck, and pressure-induced sensor attenuation. At the same time, the smart-multiple method could reconcile the reading with model estimations that are closer to expected values.

#### 5.1.2 Insulin absorption and delivery

Exogenous insulin from closed-loop APD needs to reach the maximum glucose-lowering capacity of approximately 1.5–2 h for subcutaneous administration. A high level of blood glucose has to be normalized during closed-loop delivery. The compatibility of insulin and glucagon also has to be determined in dual-hormone therapy to minimize the possibility of hypoglycemia [72]. Several models have been developed to assess insulin absorption and delivery. The observable glucose-insulin (OGI) dynamic model is one of them. This approach was used to analyze glucose regulation, glucose transport, and insulin absorption. This model features steps to personalize parameters in real-time and is proven to be able to analyze glucose dynamics *in silico* [73]. A combination of the Compartmental Model (CM) and Self Organizing Map (SOM) can also be applied for this purpose. Parameters measured included insulin infusion rate, the number of carbohydrates that had been digested, and sugar levels before the patient used the AP system. Measurements are based on glucose absorption and insulin kinetics simulated by previous glucose measurements. This method was successful in analyzing the metabolic behavior of patients with T1D [74]. Real-time state prediction of glucose and insulin levels can be done using Bergman's non-linear minimal model. This method observes conditions (plasma insulin) and parameters (glucose sensitivity) to obtain a dynamical model [75]. If you want to measure accurately, the plasma insulin concentration (PIC) estimator can be an option. This method combines the Kalman filtering algorithm and Hovorka's glucose-insulin approach. This combination model successfully predicts PIC levels in real-time based on the processing of clinical data containing significant disturbances [76].

#### 5.1.3 Control algorithms

The most widely used control algorithms are model predictive control (MPC), proportional integral derivative (PID), and fuzzy logic (FL) controls. The controller must have the ability to handle mealtime insulin boluses in a proper term or manner. The algorithms must update the state vector when the user ingests a meal or when the insulin boluses are delivered. Thus, the bolus and meal change the predicted blood glucose levels and influence the optimum profile of insulin [77]. Bequette has critically assessed various control algorithms and concluded that MPC offers several advantages in controlling the glucose level of T1D patients owing to its flexibility to deal with many challenges in AP [78]. For instance, when a meal disturbance is detected, MPC could modify the desired glucose value (setpoint), rather than only being stuck at a constant setpoint. What is more, the MPC algorithm may also recognize that the increasing value of glucose absorption into the circulation will not continue indefinitely in the future. Thus, the insulin infusion can be decreased at any time for avoiding hypoglycemic events. Furthermore, MPC algorithm may be combined with a health monitoring system (HMS) and zone controller to prevent hypoglycemia events by sending a short and multimedia message service [79]. This system has been tested to control glucose levels upon unannounced meals and exercise. The clinical result was promising, however, there is still some limitation in this study, i.e., the meal size was very small. More importantly, in a closed-loop AP system, the degradation performance must also be carefully assessed. Usually, the degradation comes from model deficiencies, poor control design parameters, and inappropriate constraints [80]. Thus, the performance assessment of control algorithms is becoming critical. Recently, a control-performance assessment algorithm has been developed, which incorporates online learning to identify the behaviors and disturbance patterns from the historical data [81].

The adaptive control is a powerful technique for controlling the complexity and variability of the dynamics of blood glucose concentration over time. Several adaptive techniques are developed to overcome these most crucial parameters of AP including (1) Run-to-run (R2R): implementing a carbohydrate to insulin ratio (CR) during the day and a subcutaneous basal insulin delivery at night where the AP performance index is calculated based on data obtained from CGM [82]; (2) Minimum variance (MV) controller: reducing the deviation of the controlled signal around the desired value and can adapt to changes in system parameters. The initial experimental trial of an AP with a predictive MV controller was carried out by Pagurek et al. [83]. A systolic pump infused the glucose solution into a vein in one arm of the study subject and a volume of blood was drawn continuously from the other arm and monitored with an auto-analyzer to determine the glucose concentration; (3) Self-Tuning Regulator: This adaptive control is developed by Astrom and Wittenmark [84]. This controller is the extension of the MV control, which is better to improve the process of identifying unknown parameters in the initial MV model. Sarti et al. [85] developed and implemented a self-tuning control algorithm for 300 simulations of insulin concentration gain with different sampling time intervals where the results obtained were more realistic than other control methods.

Recently, Hajizadeh et al. utilized machine learning techniques and patient historical data to construct personalized multivariable multimodule artificial pancreas (PMM-AP) [86]. Here, machine learning is used to modify several vital parameters, such as controller-set points and the objective function weight. These parameters are adjusted in advance to avoid meal and exercise disturbance and importantly the parameters are defined adaptively to accommodate various situations faced by T1D patients. Here, the recursive subspace-based system identification is used in combination with PIC and CGM to give better dynamic behavior of glucose variation in the patient's body over wide time ranges. Furthermore, Li et al. constructed the control algorithm with an adaptive feedback controller to update the controller parameters [87]. Interestingly, adaptive control algorithms have been successfully applied to dual-hormone AP [88]. The simulation study on three virtual patients revealed that the MPC algorithm showed promising results within a daily variation in the model parameter. Furthermore, Resalat et al. introduced a new insulin sensitivity adaptation (ISA) algorithm and adaptive learning postprandial hypoglycemia prevention (ALPHA) algorithm [89]. By combining ISA and ALPHA into MPC, the control algorithm can be used to adapt to both non-meal and postprandial periods.

### 5.2 Physiological condition of the users

On the other hand, based on the user perspective (in this case, individuals with T1D) several parameters need to be evaluated such as:

#### 5.2.1 Psychosocial assessment

The psychosocial assessment is also importantly conducted to investigate the main effect caused by the close-loop APD for the user. This assessment includes some variables such as acceptance and usage of APD, fear of hypoglycemia, and satisfaction level of APD and its treatment. These assessments can be conducted by employing the diabetes technology questionnaire and interviews with the user of closed-loop APD. The outcome of this evaluation is based on personal convenience, benefit, perceived usefulness, and perceived ease of closed-loop APD usage. A higher score can represent higher acceptance from the users [90]. Several approaches have been used to assess psychosocial factors in patients. Wristband Biosignals is a method that can be used to evaluate the presence of acute psychological stress (APS) in patients. This approach measures psychological stress signals using a classification algorithm that can distinguish physical activity (PA) from APS. The lower the APS number, the more acceptable AP systems are [91]. This approach has also been developed using machine learning which can estimate the intensity and type of APS and PA either simultaneously or individually [92].

#### 5.2.2 Individual variability

Every individual with T1D has their inter-subject variability to factors affecting insulin sensitivity such as age, gender, body weight, body height, physical condition, and lifestyle (smoking and drinking alcohol). This can influence insulin absorption and action within users [93]. An adaptive control system in close-loop APD may be required for significant variability in insulin compensation. Various studies have been demonstrated the feasibility of adaptive control systems for normalizing glycemia levels from any initial glucose levels. For instance, Pinsker et al. conducted the study by employing personalized MPC as a control algorithm [29]. The result showed that the algorithm performed well even after challenging with a 65-g unannounced meal. The personalized MPC algorithm showed 74.4% mean time in the range 70–80 mg/dL (safe glucose range). Furthermore, Dassau et al. did the clinical evaluation of a personalized APD by using multiparametric MPC (mpMPC) [94]. Here, the fully automated APD based on mpMPC was coupled with insulin on board (IOB) as a safety algorithm. The calculation of IOB was used to prevent overdosing caused by the administration of previous insulin. The result showed that the fully automated APD had good glucose control 70% of the time in the near-normal range (80–180 mg/dL). Despite the results of glycaemic control facilitated by mpMPC-IOB showing promising results, there is a problem arising from IOB calculation for determining insulin concentration i.e., the diurnal variation in the insulin diffusion, absorption, and utilization. These parameters need to be carefully considered as there are important for the personalization of APD. As a breakthrough, Hajizadeh et al. used the estimated plasma insulin concentration (PIC) to design a predictive control algorithm that is dynamically constrained by previously estimated PIC and thus explicitly accounting for the insulin concentration in the bloodstream as part of the optimal control solution [95]. What is more, Hajizadeh et al. coupled APD with wearable devices to obtain physical activity signals. Therefore, personalized APD can prevent hyperglycemia after patients perform exercises [96].

#### 5.2.3 Meal absorption and profile

The amount and size of food is an essential factors for AP systems. It is used to determine the appropriate insulin bolus dose [97]. Several approaches to meal detection in the AP have been developed. One of them is a fuzzy system using CGM, which estimates the amount of carbohydrate intake. This method calculates the number of food boluses based on the ratio of insulin and carbohydrate intake. This method allows the AP system to prevent the possibility of postprandial hyperglycemia in patients automatically [98]. The predictive control model (MPC) is another method that can be implemented. This model employs an estimation algorithm based on continuous observations of the first and second derivatives of glucose. This will generate an impulse to eat which will later be converted into grams of carbohydrates that can be measured [99]. Another method is the Unscented Kalman Filter. This approach measures the cross-covariance between the difference in the rate of disturbance and the measured glucose. This is done to analyze the sensitivity and the amount of carbohydrate intake in patients. This approach successfully detects this aspect of sensitivity [100].

A more comprehensive description of the necessary tests to be conducted to determine the device's effectiveness is described in the *regulatory requirements section: APD system performance*.

## 6 Regulatory requirements

The regulatory requirements for APD are based on the document of Guidance for Industry and Food and Drug Administration (FDA) Staff: The Content of Investigational Device Exemption (IDE) and Premarket Approval (PMA) Applications for Artificial Pancreas Device Systems by 2012. This guidance consists of background, scope, device description, indication for use, APDs performance, clinical study progression, labeling, manufacturing, and post-approval study. The FDA recommends the manufacturer provide these descriptions, including (1) system level; (2) functional components of glucose monitoring; (3) functional components control algorithm and signal processing; (4) functional components of the infusion pump, and (5) functional components of the communication pathway. FDA recommends all information about individual parts of the device should be provided. Requirements for current good manufacturing practice (GMP) that are included in Quality Systems (QS) regulations are also necessary. The FDA anticipates that most APD will require post-approval studies (PAS) to better assess long-term and real-world patient performance and/or outcomes. FDA recommends the PAS protocol be developed and be submitted the protocol with the original PMA [101].

## 7 Data about the market and competition for the device

Market and competition data of closed-loop APD is based on each structural part of the device, including (1) Insulin infusion pump (IIP) and (2) Continuous glucose monitoring (CGM) devices. There are three main types of IIP that work incorporation with CGM. In the first type, the CGM delivers the value of the glucose sensor, and then it will be displayed on the IIP. In this type, no insulin dosing is provided based on the value of CGM. In the second type, the CGM sends sensor glucose value to the IIP then it can suspend insulin when the blood value achieves the low threshold limit [102]. The last type is the hybrid closed-loop APD, where the CGM delivers sensor glucose values to the IIP, then it can decide insulin dosing on basal insulin delivery from the obtained values [103].

For many current years, the insulin infusion pump has been developed notably. Some of them, work as stand-alone insulin delivery devices without a CGM as an integrated medical device. Presently, there are three available hybrid closed-loop APDs available in the market. First, Medtronic plc commercializes the automated insulin delivery system with a hybrid closed-loop APD MiniMed™ 770G at a retail price is approximately USD 899. This price is applicable as an offer when the device is upgraded from the older Medtronic MiniMed™ 670G. For reference, the approved full price regulated by the ministry of health Canada is USD 6300 [104]. This insulin pump can be integrated with Guardian™ Sensor 3 CGM, and it has been approved by the FDA and is already available in the United States and several European countries, including Belgium, Denmark, Finland, Ireland, Italy, Netherlands, Spain, Sweden, Switzerland, and the United Kingdom [105]. The second company is Tandem® Diabetes Care with t: slim X2™ insulin pump. This insulin pump can be incorporated with Dexcom G5® or G6® CGM. This insulin pump can be obtained by upgrading the older version by USD 799 for the new condition and USD 399 for refurbished units [106]. Moreover, the full price of the new unit is the cost of USD 6300 [104]. The third hybrid APD available is developed by CamDiab with the market name CamAPS FX. CamAPS FX is an android app that can be integrated with pump and CGM. CamAPS is compatible with Dana Diabecare RS® pump and Dexcom G6® CGM. Unlike the two other APDs, CamAPS requires the user to pay for an app subscription. In summary, the market data for APD is shown in Table 1.

**Table 1:** The available artificial pancreas devices in the market.

	The MiniMed™ 770G system	t:slim X2™ insulin pump with Control-IQ™ technology	CamAPS® FX with Dana Diabecare RS and Dana-i pump
Manufacturer	Medtronic plc	Tandem® Diabetes Care	CamDiab
CGM system	Guardian™ 3	Dexcom G6®	Dexcom G6®
Smartphone integration	Android and iOs	Android and iOs	Android
Price	Pump: $899 (upgrade from older version) or $6300 (Full price) Rechargeable Transmitter: $1100 (1 year warranty, may last longer) Sensors: $450 for box of 5 (35 day supply) App: $0	Pump: $799 (upgrade from older version) or $6300 (Full price) Transmitter: $300 every 90 days Sensors: $420 (30 day supply) Receiver: $0 (if using smartphone) or $380 one time purchase App: $0	Pump: $5500 Transmitter: $300 every 90 days Sensors: $420 (30 day supply) Receiver: $0 (if using smartphone) or $380 one time purchase App: • $100 (monthly subscription) • $600 (6 months subscription) • $1100 (annual subscription)
Release year	September 2020	January 2020	2020
Reference	[107, 108]	[106, 108, 109]	[108, 109, 110]

## 8 Future perspective

Improvements to the components of the closed-loop artificial pancreas system are likely to maximize performance and user comfort. The device workload is expected to be minimized with the development of the continuous glucose monitoring component with optimized accuracy, longer usage time, and fully integrated into insulin pump and smartphone (as a data receiver). In a recent development, for instance, the MD-Logic Artificial Pancreas algorithm (a fully digitalized and advanced hybrid closed-loop system) poses a promising performance in controlling user glucose levels and has been validated in clinical trials. A very fast-acting and accurate level of insulin is also needed to be fully automated based on the employed algorithms. A user-friendly interface of smartphone applications is required for the user and health care professionals to evaluate the benefits of using the device. Finally, cost-effectiveness studies are required for the health care system in taking reimbursement decisions regarding the use of this device.

## Declarations

### Author contribution statement

All authors listed have significantly contributed to the development and the writing of this article.

### Funding statement

This research did not receive any specific grant from funding agencies in the public, commercial, or not-for-profit sectors.

### Data availability statement

Data included in article/supp. material/referenced in article.

### Declaration of interest's statement

The authors declare no conflict of interest.

### Additional information

No additional information is available for this paper.
